# Transformation-induced changes in the DNA-nuclear matrix interface, revealed by high-throughput analysis of DNA halos

**DOI:** 10.1038/s41598-017-06459-7

**Published:** 2017-07-25

**Authors:** Rosemary H. C. Wilson, Dawn Coverley

**Affiliations:** 1 Department of Biology, University of York, Heslington, York, YO10 5DD UK; 2 Present Address: Sir William Dunn School of Pathology, University of Oxford, South Parks Road, Oxford, OX1 3RE UK

## Abstract

In higher eukaryotic nuclei, DNA is periodically anchored to an extraction-resistant protein structure, via matrix attachment regions. We describe a refined and accessible method to non-subjectively, rapidly and reproducibly measure both size and stability of the intervening chromatin loops, and use it to demonstrate that malignant transformation compromises the DNA-nuclear matrix interface.

## Introduction

**Figure 1:**
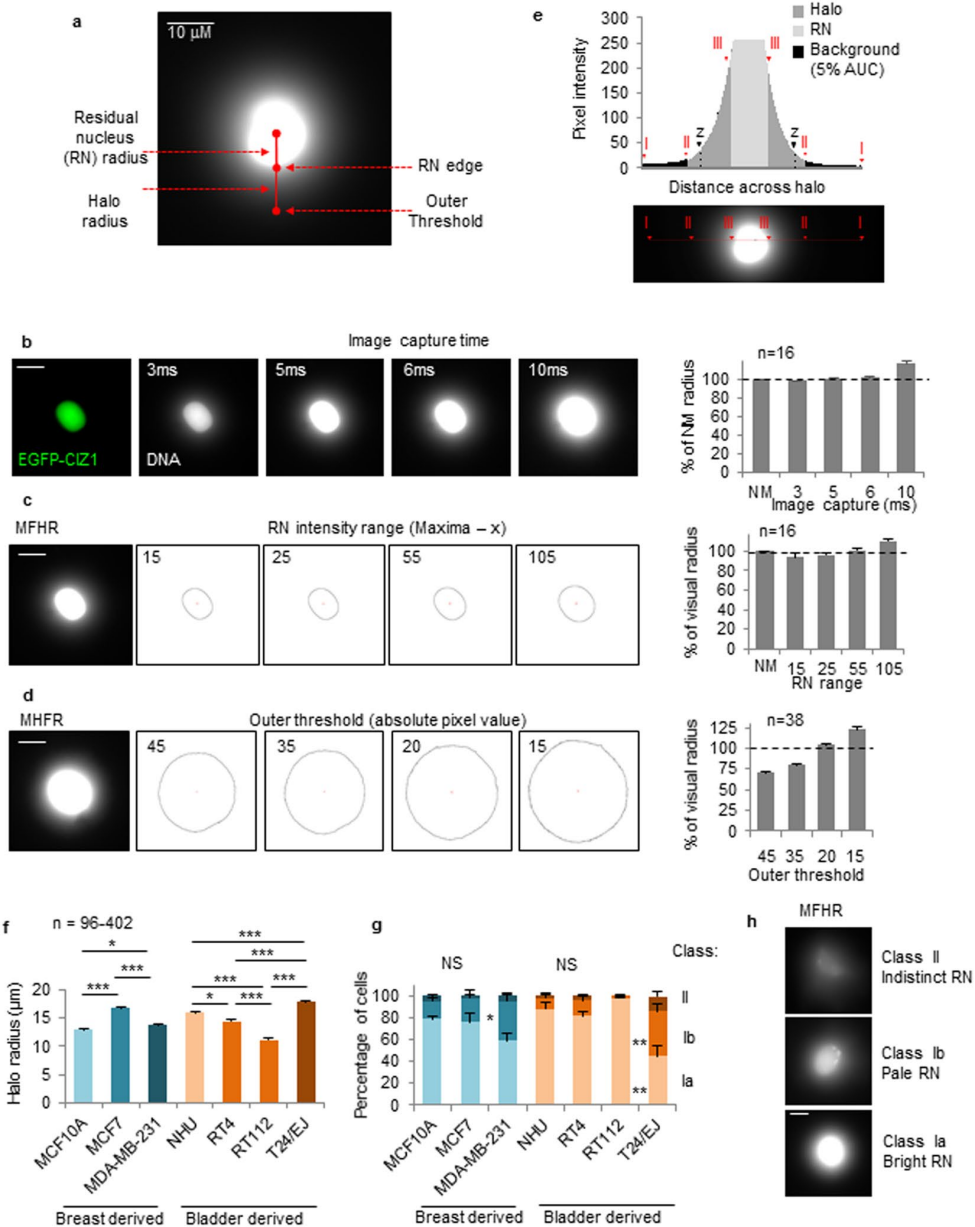
Calibration and edge determination. (**a**) Example MFHR image. (**b**) NM marker (green) and DNA in MFHR-extracted nuclei (white). Histogram shows mean RN radius as percentage of NM marker, +SEM. (**c**) RN and (**d**) outer threshold positions, determined by HIM, using indicated pixel threshold values. Histograms show mean derived radius as percentages of visually-derived values. (**e**) Pixel intensity plot across an MFHR-processed nucleus, showing outer threshold (II), RN threshold (III), and typical outer threshold estimated by eye (z). (**f**) Mean halo radius and (**g**) class distribution for indicated cell lines, +SEM. (**h**) Examples of class Ia, Ib and II MFHR-processed cells.

The three-dimensional organization of chromatin plays a central role in the regulation of nuclear functions. Organization includes periodic attachment to a biochemically-defined, extraction-resistant structure referred to as the nuclear matrix (NM), via Scaffold or Matrix Attached Regions of DNA (S/MARs). However, despite extensive analysis of S/MAR sequences and evidence that replication and transcription take place at the base of chromatin loops, the concept of a NM is not universally accepted, and has been under investigated. As a result, we have limited information on the changing relationship between chromatin and the NM during development and disease^1^ and a very limited understanding of the functional significance of pathology-associated changes in nuclear structure^2^. The Maximum Fluorescence Halo Radius (MFHR) method^3^ has the potential to reveal information about the relative role of loop-base attachments in different cell types. It allows visualization and measurement of loops and the residual nucleus (RN), after unpackaging by removal of histones and other associated proteins, whilst maintaining NM attachment (Fig. 1a). It has been used to investigate average chromatin loop size^4, 5^, NM attachment of individual genes^6, 7^ the effect of replication rate^3, 8, 9^, and of knockdown of specific proteins^10, 11^. However, its full potential to follow changes in chromatin loops has not been realized because traditional methods for analysis of halo images are labor intensive, rely on subjective visual assessment of radius measurements and are vulnerable to user variability. Moreover, typically only defined halo structures are quantified which omits important information about populations of cells. We have developed an accessible MFHR image analysis method and established a straightforward procedure to set threshold parameters. Halo Image Macro (HIM), used with NIH ImageJ^12^, enables rapid, non-subjective quantification of average DNA loop size in populations, calculated from the shape of the whole DNA halo. We have also developed related assays that report on loop stability (Supplementary Table 1), and applied these to reveal oncogene-induced changes in the chromatin-NM interface. MFHR is a conceptually simple but technically challenging method, which has the potential to return valuable data about an under-investigated area of cell biology.

## Results

### Establishment of MFHR halo edge thresholds for image analysis

Accurate quantification of mean chromatin loop size is based on establishment of two ‘edges’; the outer threshold of the halo and the residual nucleus (RN) (Fig. 1a). Firstly we establish the correct exposure for MFHR image capture using RN size, by calibrating settings to the area occupied by the NM. We use a GFP-tagged NM protein (EGFP-CIZ1 C-term274^13^) to mark the NM, and set MFHR image exposure. For our apparatus 5 ms returns a mean value for RN radius that is 100.3% of CIZ1 NM radius (Fig. 1b).

So that the pixel intensity threshold that specifies the edge of the RN is compatible with the wide range of intensities within a population of MFHR processed cells (Supplementary Fig. 1a), we use an RN threshold that is related to individual image maxima. To select the RN threshold, RN radius measurements returned by HIM with a range of settings are compared to those generated by visual assessment of the RN boundary, for a training set of images. In the following experiments we use an RN equal to the local maxima less 55 (x = 55), which returned 101% of visual estimates of the training set (Fig. 1c, Supplementary Fig. 1b).

The pixel intensity for the outer threshold is more difficult to determine visually, and is subject to both user and screen variability (Supplementary Fig. 1c). Unless indicated, we use an absolute pixel intensity threshold of 15, which captures ≥95% of the area under the curve and confidently includes the outermost DNA loop edge across a population of cells (Fig. 1d,e, Supplementary Fig. 1d).

With a fixed image capture time, established RN and outer thresholds, HIM is capable of systematic comparison of multiple large data sets and automatically returns linked measurements for RN area and total area, for batches of submitted images (Supplementary Fig. 1e). Straightforward calculations convert these to halo radius (see User Guide).

### Cell lines derived from high grade tumors exhibit destabilized halos

Comparing halo size within a set of breast and bladder derived cell lines revealed considerable variation between populations (Fig. 1f). Moreover, all cell types gave rise to two classes of product, differing in their RN. Class I have defined RNs while class II have a poorly defined RN and fail to be measured by HIM x55/15 (Fig. 1g,h). A bright, well defined RN reflects greater retention of DNA within the residual nucleus and implies strong DNA:NM attachments that withstand the extraction process. Class I cells can be further divided into Ia (bright RN) or Ib (pale RN) using a specialized HIM (Supplementary Fig. 2). In fact most published MFHR analyses do not comment on class differences, measuring only defined and regular MFHR entities (and showing only class Ia cells), thereby under-reporting differences between populations.

Notably class distribution, determined by RN HIM, is significantly different between the poorly differentiated breast and bladder cancer cell lines MDA-MB-231 and T24/EJ, compared to lower grade tumor-derived lines, or cells derived from normal urothelial tissue. This manifests as increased class Ib and class II cells (Fig. 1g), and suggests that destabilization of the DNA:NM interface is a feature of malignant transformation.

### Effect of introduced oncogenes

**Figure 2:**
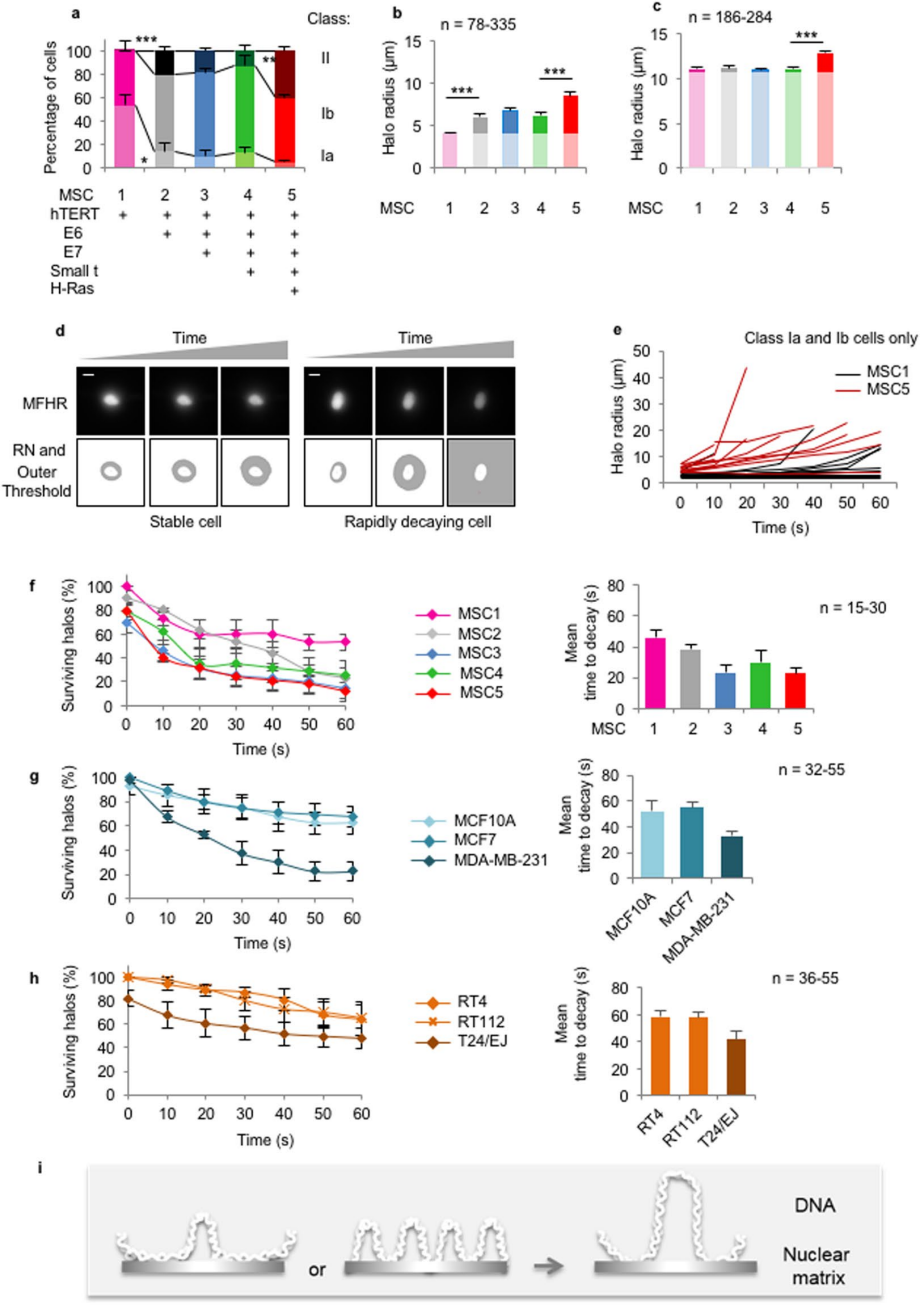
Transformation-induced changes in chromatin loop size and stability. (**a**) Class distribution for Mesenchymal Stem Cell (MSC) series, with introduction of the indicated oncogenes^14^. (**b**) Mean halo radius using HIM x75/x180 or (**c**) HIM x75/15. (**d**) Example MFHR stability over time, showing edges determined by HIM x75/x180. (**e**) Halo measurements over 60 s time-course for individual MSC1 and MSC5 cells. (**f**) Stability measurements plotted as percentage of surviving halos (left), and average time to decay (right). (**g**) and (**h**), as in f, for breast and bladder-derived cell lines respectively. (**i**) Possible effects of transformation on DNA:NM interface.

To interrogate this under more defined conditions we used an isogenic Mesenchymal Stem Cell (MSC) series, with sequential introduction of five well-characterized oncogenes^14^ (Figs 2 and Supplementary Fig. 3a). Variation between the five populations necessitated careful selection of HIM thresholds to support measurement of cells over the whole series under the same conditions (Supplementary Fig. 3). Range from local maxima was selected for both RN and outer threshold (x75/x180), generating data that reflect both chromatin loop size and RN intensity. This revealed significant shifts toward larger and more diffuse halos after expression of HPV-E6 (50% increase in mean halo size and 4 fold decrease in class Ia cells), and again after expression of H-Ras (111% increase in size and 3 fold increase in class II cells), compared to the starting population (Fig. 2a,b). In contrast, neither HPV-E7, nor SV40 small-t antigen had a measureable impact on either parameter. A second set of HIM parameters that use an absolute outer threshold (x75/15), making size determination independent of RN intensity, confirmed that halo radius is affected by H-Ras expression (Fig. 2c). However, the effect of E6 was not observed, suggesting that it likely reflects a complex effect on structure of the NM itself (RN). Consistent with this, larger RN sizes were recorded following E6 expression (Supplementary Fig. 3h).

### Stability of MFHR halos in five-member isogenic transformation series

DNA halos are unstable structures so that class I halos transition to class II halos over time. The wavelength used to image ethidium bromide-stained halos (546 nm excitation) induces DNA damage^15–18^, by introducing both double and single strand breaks. Cells that start with a bright RN and have more DNA associated to the nuclear matrix (class Ia) appear to withstand more stochastic damage before DNA is released and can diffuse away. This instability limits image capture time but, by applying HIM with an outer threshold related to the local maxima, it also offers a means to measure dynamic changes in halo stability (Fig. 2d,e). As DNA is fragmented and lost, the intensity drops until a threshold is reached when the outer edge of a halo can no longer be measured (<180 for HIM x75/x180), and a cell ‘fails’ to return a value (Fig. 2d). By comparing a series of images captured over 60 s, we can classify halos into stable or rapidly decaying entities, allowing time to decay and percentage of surviving halos to be plotted. This shows that halos are much more stable in MSC1 cells than in MSC5 cells (Fig. 2e,f). Similar results were obtained for high-grade tumour cell lines MDA-MB-231 and T24/EJ (HIM x55/x240), compared to lower grade cells (Fig. 2g,h). Partial release of loop DNA from the NM, and differential stability, has been noted previously but has not been quantified^19^.

## Discussion

The isogenic MSC cell series used here are characterised by acquisition of malignant phenotypes upon expression of defined oncogenes. Most notably, anchorage independent growth and tumour formation in mice both increase dramatically upon introduction of the GTPase H-Ras into MSC4 cells (which already harbour HPV-E6, HPVE7 and small t-antigen) to generate MSC5 cells^14^. In our experiments introduction of H-RAS, which bypasses growth factor regulation, initiates a transition toward larger halos, identifying changes in S/MAR recruitment as co-regulated with these malignant characteristics. Induced expression of H-Ras has been previously linked with changes in the protein component of the NM and DNA supercoiling^20^, offering some precedent for these findings. We also show that HPV-E6 which inactivates the tumor suppressor p53, initiates a separate transition in size, stability and class, all of which suggest fewer or shorter interface between DNA and the NM. Both HPV E6 protein and viral DNA are found associated with the NM^21^, however, there is no further evidence to support a direct link with stability or structure of the nucleus.

Neither H-Ras or E6 are reported to increased proliferation rate in this MSC series, compared to their parent line (though introduction of t-antigen to generate MSC4 did^14^), arguing that DNA:NM stability is not directly related to proliferation rate. This is important because cell cycle distribution might be expected to impact on the DNA:NM interface when replication origins are recruited to the NM during initiation of DNA replication^1^. In fact published work^22^, and our own analysis (not shown), which compared unsynchronised cells, and G1 or S-phase cells did not support global MFHR loop size differences during the mitotic cell cycle.

There has been some genome wide analysis of NM-attached DNA in two of the breast-derived lines used here^23^, which reports differences that compare well with our results. Specifically, in MCF10A cells NM-DNA was more likely to map to gene rich regions and to expressed genes than in MDA-MB-231, where NM-DNA was enriched in non-expressed genes. However, in both lines H3K4me3, H3K27me3 and H3K9me2 were appropriately associated with expressed and non-expressed genes, suggesting that NM attachment is not closely related to epigenetic landscape. The relationship may not be clear cut however, because analysis of methylation status of LINE1 and Sat2 elements in the MSC1-5 cell series revealed hypomethylation in the MSC5 line^24^. Though a direct correlation between this study of DNA methylation and our study of NM-attachment cannot be drawn, DNA methylation of specific S/MARs has been reported to affect function^25^.

The molecular basis for the increases in loop size and decrease in stability remains to be determined. Clearly, global changes in transcription rate, reported in cancer cells^26^, could affect the DNA:NM interface, and there are numerous published examples of tumour-associated changes in nuclear matrix protein composition^2, 27–30^ or sub-nuclear architecture at the level of domain organization^2^. Moreover, there are specific examples of diseases that are associated with loss of S/MAR sequences^31^. Despite the strong correlation between degeneration of nuclear architecture and cancer, there is little evidence that distinguishes cause and effect. Loss of stabilizing architecture could facilitate loss of cellular identity through relaxation of spatial control over gene expression^30^, or reduced anchorage of chromatin loops might directly support emergence of genome instability through inaccurate repair of strand breaks. Alternatively, loss of stable structure is a consequence of cancer-associated changes in gene expression. Here we present evidence that introduction of a transforming oncogene *causes* (directly or indirectly) disruption of the DNA:NM interface. Moreover, the approach described here offers a well-defined methodology with which to further dissect malignancy-associated changes in nuclear structure, starting with the previously unrecognised trigger, H-Ras.

## Methods

### Cells and cell culture

Cells were obtained from ATCC or JCRB cell banks and grown on glass coverslips to 50–70% confluence, as recommended. Sequentially transformed mesenchymal stem cells^14^ (MSC) were kindly provided by Dr Juan Funes and Prof Chris Boshoff, and normal human urothelial cells (NHU), derived from tissue biopsies^32^, by Edward Bowen and Prof Jennifer Southgate. Breast cell lines were derived from normal breast tissue (MCF10A^33^), low grade breast cancer (MCF7^34^) or oestrogen receptor negative metastatic breast cancer (MDA-MB-231^35^). Bladder cell lines were from metastatic bladder cancer (T24/EJ^36^) or lower grade bladder cancers (RT4 and RT112^36^).

### Maximum Fluorescence Halo Radius extraction method

The ethidium bromide MFHR method has been described previously^4, 9^. For the analysis performed here, the following changes were made: 0.25% Igepal (NP40 substitute, Fluka 56741) was used instead of 0.5% NP40, and a development incubation was included (5 mins in the dark) to enable halos to reach a stable expanded state prior to imaging. All incubations were performed on ice using ice-cold buffers. Briefly, cells on coverslips were washed in D-PBS followed by 1 min incubation in detergent extraction buffer (0.25% Igepal, 10 mM MgCl_2_, 0.5 mM CaCl_2_, 50 mM Tris-Cl pH 7.5). Coverslips were then incubated for 30 s in series in each of 0.5 M NaCl, 1.0 M NaCl and 1.5 M NaCl extraction buffers, followed by 2.0 M NaCl extraction buffer (all made up in 0.2 mM MgCl_2_, 10 mM Tris-Cl pH 7.5). The last incubation lasts for 2 min, including 1 min exposure to 240 nm UV treatment, and is done in the presence of 100 μg/ml ethidium bromide. Coverslips were then mounted on slides, sealed with nail varnish and matured in the dark for 5 min.

### Microscopy and image capture

Halos were imaged using a Zeiss Axiovert 200 M fluorescent microscope using Zeiss filter set 15 (Excitation filter: band pass 546/12 nm, Emission filters: beam splitter 580 nm and emission long pass 590 nm) and AxioCam HRm digital camera with Openlab software (PerkinElmer). Images were acquired using constant exposure (5 ms unless stated otherwise), taking care to image only ‘fresh’ fields that were not previously viewed. 50–80 images (RGB 8-bit, file size 1388 × 1040 pixels) were acquired from each sample, within 10 min of preparation. Raw Openlab LIFF files were saved for reference but files were transferred with no additional processing as RGB 8-bit TIFF files for analysis in ImageJ.

### Visual size estimates

For analysis by eye, MFHR files were opened in ImageJ 1.46 for Mac OS X (NIH). Halo areas were calculated using the ‘polygon’ function to draw around the whole of the visible RN and halo (Supplementary Fig. 1b), quantified using the ‘measure’ function, and radius measurements derived using the formula in HIM User Guide. Alternatively halo radius measurements were estimated using the ‘line’ function to draw a vertical line from the outer edge of the RN to the outer edge of the visible halo, at the lowest position in each image (Supplementary Fig. 1b), followed by the ‘measure’ function. Irregularities in structure have significantly more impact when using a single radius measurement.

### Halo Image Macro (HIM) analysis

Image files were processed using ImageJ macro HIM to calculate average halo radius, class percentages or stability of halos as described. HIM versions for each type of analysis are indicated in main text and see Supplementary Table 1. HIM set up and validation is described in results and supplementary information. Briefly, HIM defines RN and outer halo edges, creating an ROI for both regions. HIM matches RN and outer regions where possible using halo positional information. Output is a RN threshold analysis picture and outer threshold analysis picture for each image, and a .csv file containing RN and outer area measurements. Users then derive the halo radius. MSC cell lines were processed for halo radius size using HIM x75/15 or HIM x75/x180 as indicated. Other cell lines were processed for halo radius analysis using HIM x55/15.

### Using HIM to calculate halo class distribution

In addition to a range of halo diameters, populations of cells processed by MFHR return two classes of product (Fig. 1h). Class I have defined RNs and class II have an ill-defined RN with poor structure. Class I is further subdivided into Ia (bright) where >50% of the returned RN from local maxima is above 220 intensity and Ib (pale) where <50% of their RN above 220 intensity. Visual designation can be subject to user variability (not shown), therefore we designed a specialised classification HIM (RN HIM) which uses RN intensity to non-subjectively classify halos into class Ia, Ib or II (Supplementary Figs 2, 3, Supplementary Table 1). MSC cell lines were processed for classification analysis using HIM RNx75/RN220, other cell lines were processed using HIM RNx55/RN220.

### Using HIM to measure stability

For halo size the time each halo is exposed to light before/during imaging is 1–2 seconds, therefore the opportunity for halo degradation is minimal. In contrast, for stability measurements, halos are imaged through a 60 second window to reveal time-dependent decay, as seen in Fig. 2d–h. A series of images collected over time can be used to quantify stability and rate of halo decay. We typically collect 7 images of individual cells from 0 s to 60 s at 10 s intervals using 546 nm light (see microscopy and image capture), typically from 8 cells per coverslip for at least four coverslips. Users should decide if they wish to sample from the whole population or from one class. After curation (identification of whole image ROIs derived from failed cells), we plotted surviving halos as percentage of the population for each time point. Alternatively, halo failure time can be used to plot average time to decay. Where halos did not fail, and remained measureable at 60 s, they were given a fail time of 70 s. MSC cell lines were processed using HIM x75/x180, other cell lines were processed using HIM x55/x240.

### Western blotting

Whole cell lysates were generated from asynchronously growing MSC cells, cultured as recommended^14^. Equal amount of sample were run on SDS-PAGE and transferred to 0.1 µm nitrocellulose before being probed for introduced oncogenes, to validate MSC cell line identity. Antibodies used were mouse anti-PCNA (PC10, abcam), rabbit anti-HRAS (18295-1-AP, Proteintech), E6 (N17, Santa Cruz) and mouse anti-actin (A4700, Sigma) as a loading control.

### Sample numbers and statistics

Mean halo radius data, classification data and stability data were generated from image sets derived from four coverslips, comprising two technical replicates from each of two biological replicates. Stability data are presented as percentage of surviving halos at each time point or average time to decay. These were typically generated from 32 cells, comprising 8 cells (each followed for 60 s) from each coverslip. All error bars are SEM. Powers were calculated using Student’s t-test, where *p,0.05, **p < 0.005, ***p < 0.0005.

## Electronic supplementary material

Supplementary information

Supplementary information

Supplementary information

Supplementary information

Supplementary information

Supplementary information

Supplementary information
