# Supplementary material for: Transformation-induced changes in the DNA-nuclear matrix interface, revealed by high-throughput analysis of DNA halos
Source: Sci Rep. 2017 Jul 25;7:6475. doi: 10.1038/s41598-017-06459-7 (PMC5526987; doi:10.1038/s41598-017-06459-7)
Supplement: Supplementary file 1 — Supplementary information [file 41598_2017_6459_MOESM1_ESM.pdf]

Supplementary Information for:

Transformation-induced changes in the DNA-nuclear matrix interface, revealed by high-throughput analysis of DNA halos.

Rosemary H.C. Wilson[1, 2, *] and Dawn Coverley[1]

[1] Department of Biology, University of York, Heslington, York, UK, YO10 5DD
[2] Current address: Sir William Dunn School of Pathology, University of Oxford, South Parks Road, Oxford, UK, OX1 3RE

*Correspondence: rosemary.wilson@path.ox.ac.uk

Legends to supplementary figures

Supplementary figure 1: Validation of HIM a) Pixel intensity plots across example MFHR images, showing intensities across the whole of the image (upper), flattening out over the higher intensity RNs. The lower plot shows captured RNs after application of local maxima less 55 (x55) threshold. Plots show representative sample class Ia (RN reaching maximal 255 intensity) and class Ib (RN maxima less than 255) images. b) Schematic shows RN and halo boundaries. c) Six data sets extracted from the same population of images, expressed as frequency distribution of halo radius measurements. Results were generated using visual or HIM analysis as indicated, or by different users and screens. d) The effect of RN and outer threshold settings on average halo radius for the same population of images, showing little variation associated with changes in RN threshold intensity setting, and a more dramatic dependence on outer threshold setting. Our default HIM setting of x55/15 is shown in black. e) Schematic shows images produced by ImageJ during HIM process. HIM selects an ROI for the input image (left), using either a threshold related to the local maxima (RN, top set) or an absolute pixel intensity threshold (outer threshold, lower set). The ROI edge is closed and set to 255 (only required for threshold related to local maxima). HIM then measures the ROI area and produces a numbered ROI image (analyse particles). f) Average RN radius, halo radius and total radius for a sample data set of 3T3 cells, shown as mean (upper), or frequency distribution (lower). g) Graphs show pixel intensity across an MFHR treated nucleus (left) and one that was treated only up to 0.5M NaCl, so that histones are not extracted and DNA does not expand beyond the RN. Boundaries selected by HIM x55/15 are indicated.

Supplementary figure 2: HIM based classification of class Ia and Ib halos a) Left: Histogram shows the percentage of the RN area captured by HIM x55/15, that is also captured using an absolute intensity value for RN threshold (as indicated), for a representative set of 3T3 images. This effectively separates class Ia and Ib cells, because for Ia cells the two measurements are similar, and the % is close to 100. RN 220 was selected for separation of class Ia and Ib cells and validated (using HIM RNx55/RN220) by comparing results to visual classification of a 3T3 image training dataset (right). The two outputs were aligned with no 'false Ib' or 'false Ia' (where HIM classification does not match visual assessment). b) Histogram shows mean RN, halo and total radius measurements for class Ia and class Ib 3T3 cells, using x55/15. c) Histogram shows mean halo radius for class Ia, class Ib or both class Ia and b for a set of non-cancer cell lines, using x55/15. The same trend is observed when independent of class used. d) Histogram shows classification for non-cancer cell lines used in c.

Supplementary figure 3: Customization of HIM for analysis of linked set of MSC lines a) Western blot validation of MSC lines 1-5 showing introduced oncogenes as indicated. b) Graph shows RN radius calculated by HIM using the indicated RN thresholds (local maxima less x) for a sample set of MSC1 (n=100) and MSC3 (n=66) images. Values are plotted as percentage of RN radius calculated using visual assessment. c) Histogram shows average halo radius for MSC1 and 3 cells, with the indicated HIM threshold settings. MSC1 and 3 return a difference regardless of settings. We prefer x75/x180 as this allows a greater proportion of cells to return a measurement. For MSC1, n=41 with x55/x240, n=44 with x75/x240, n=83 with x75/x180. For MSC3, n=2 with x55/x240, n=2 with x75/x240, n=16 with x75/x180. d) Graph shows percentage of MSC1 and 3 cells measureable, with the indicated outer thresholds (using constant RN of x75). At x230 and x240 most MSC3 cells fail to return a value. For analysis of the full MSC series, shown in Fig. 2b, x75/x180 was applied. e) Establishing settings for classification of Ia and Ib by HIM. Histogram shows the percentage of the captured RN (local maxima less 75) that is also captured when using the indicated absolute intensities for RN, for a representative set of MSC1 images. f) Histogram shows classification of MSC1 images using the indicated HIM parameters compared to visual assessment of the training set. Images that were visually scored to be class Ia but by HIM were scored to be class Ib are defined as ‘false Ib’, and those visually scored to be Ib but by HIM scored as Ia are defined as ‘false Ia’. RN 220 most accurately separates the classes. g) As e) for representative MSC1, MSC3 and MSC5 images, using HIM RNx75/RN220. h) Histogram shows mean RN radius (for class Ia and b) across the MSC series using HIMx75/15, also used to generate the data in Fig. 2c.

Supplementary figure 4: Analysis of variance for MSC lines a) Frequency distribution graph shows data for all five MSC cell lines, each combined from four replicate experiments. b) Frequency distribution graph shows individual replicates for MSC1 and MSC2. c) As b for MSC4 and MSC5. d) As b for MSC2 and MSC4. e) Table shows ANOVA results for the indicated groups. f) Histograms show average halo size for MSC1 and MSC4 (left) and MSC4 and MSC5 (right) when pairs were processed together and imaged on the same slide at the same time. Data collected from three replicates for each pair of cell lines. Error bars are SEM, * $p<0.05$, *** $p<0.0005$. No significant difference between MSC4 collected with MSC1 or MSC4 collected with MSC5.

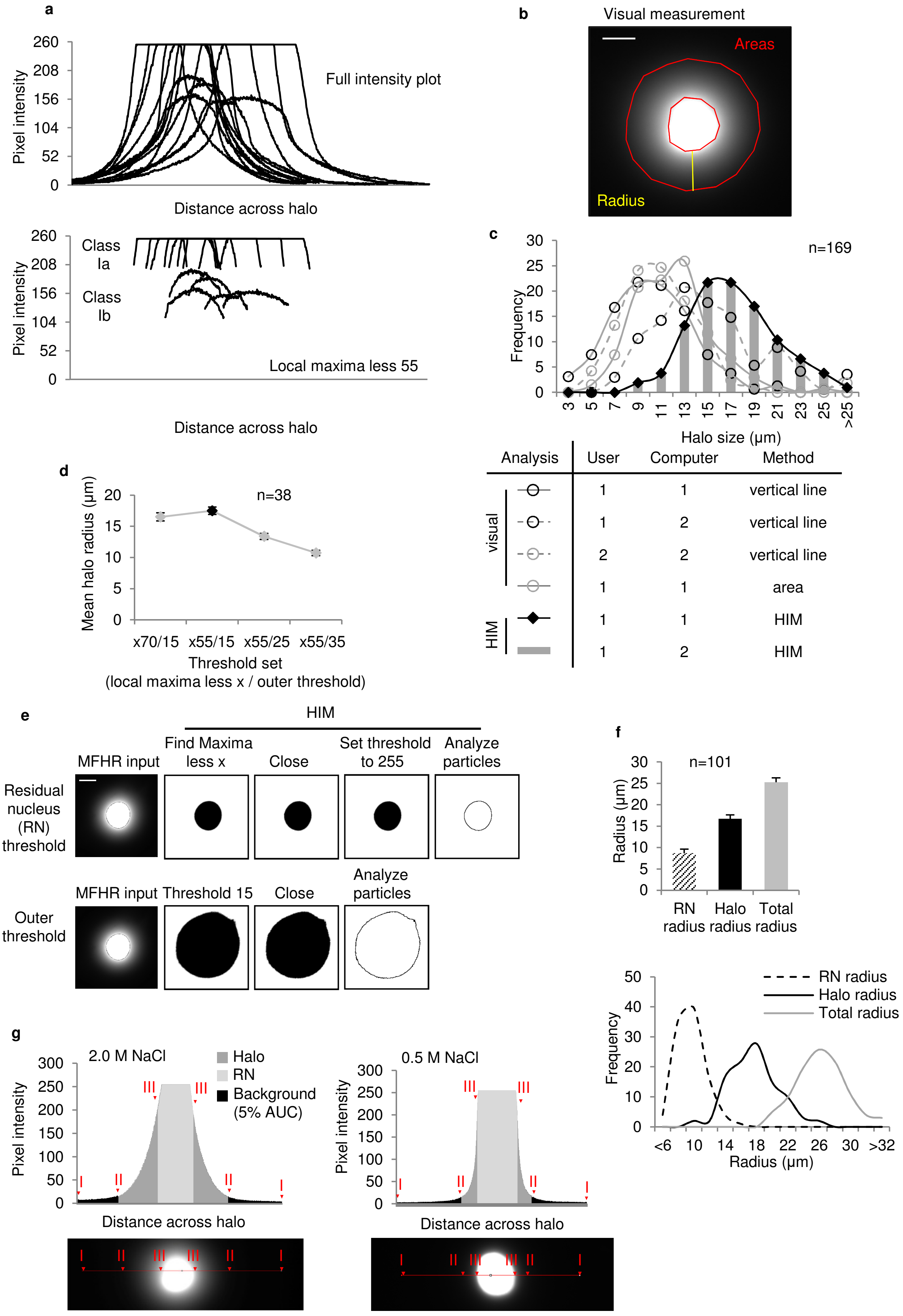

Supplementary Figure 1

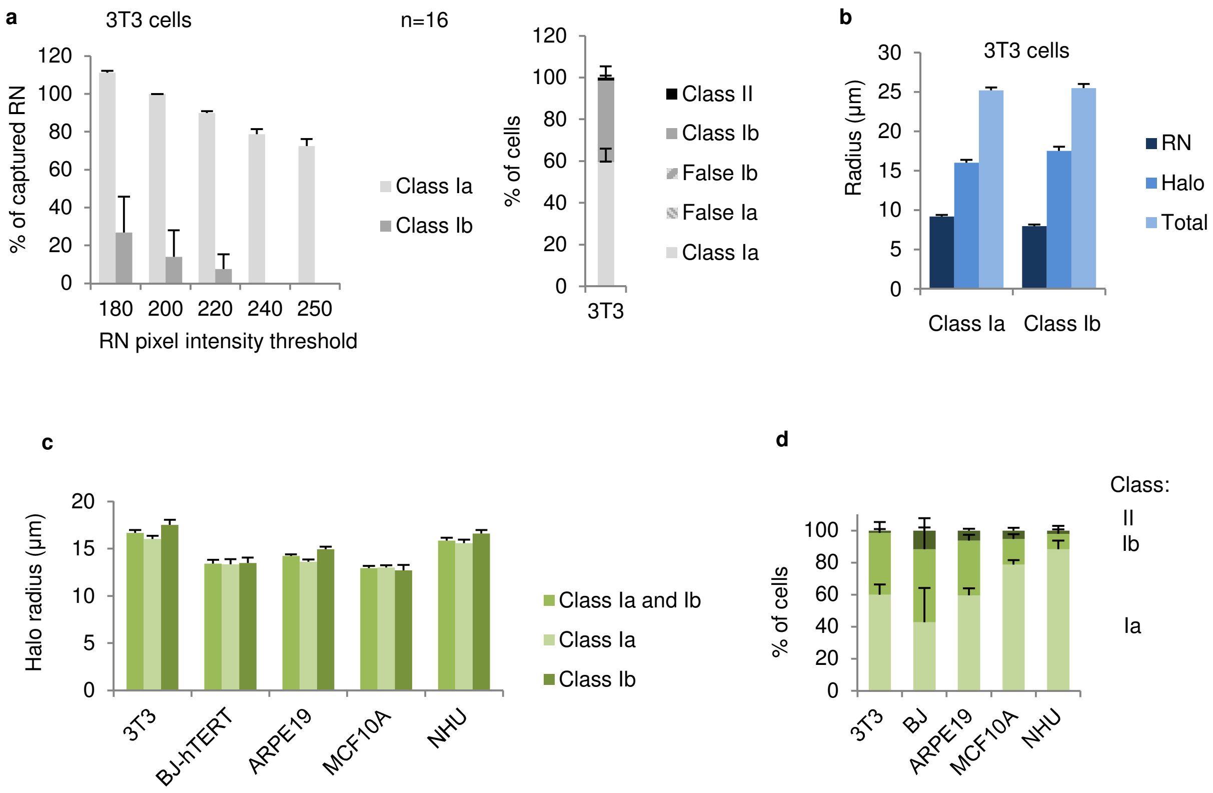

Supplementary Figure 2

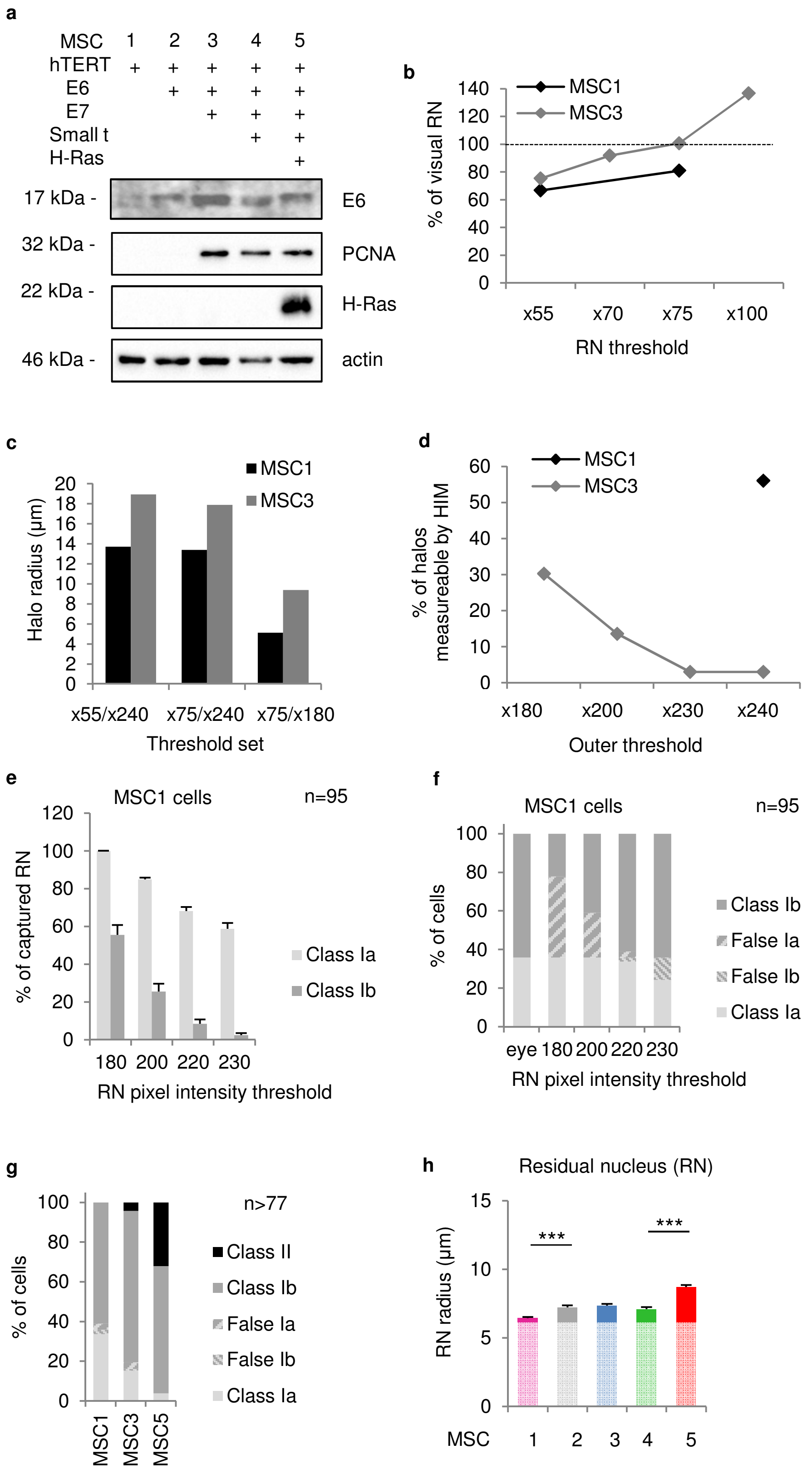

Supplementary Figure 3

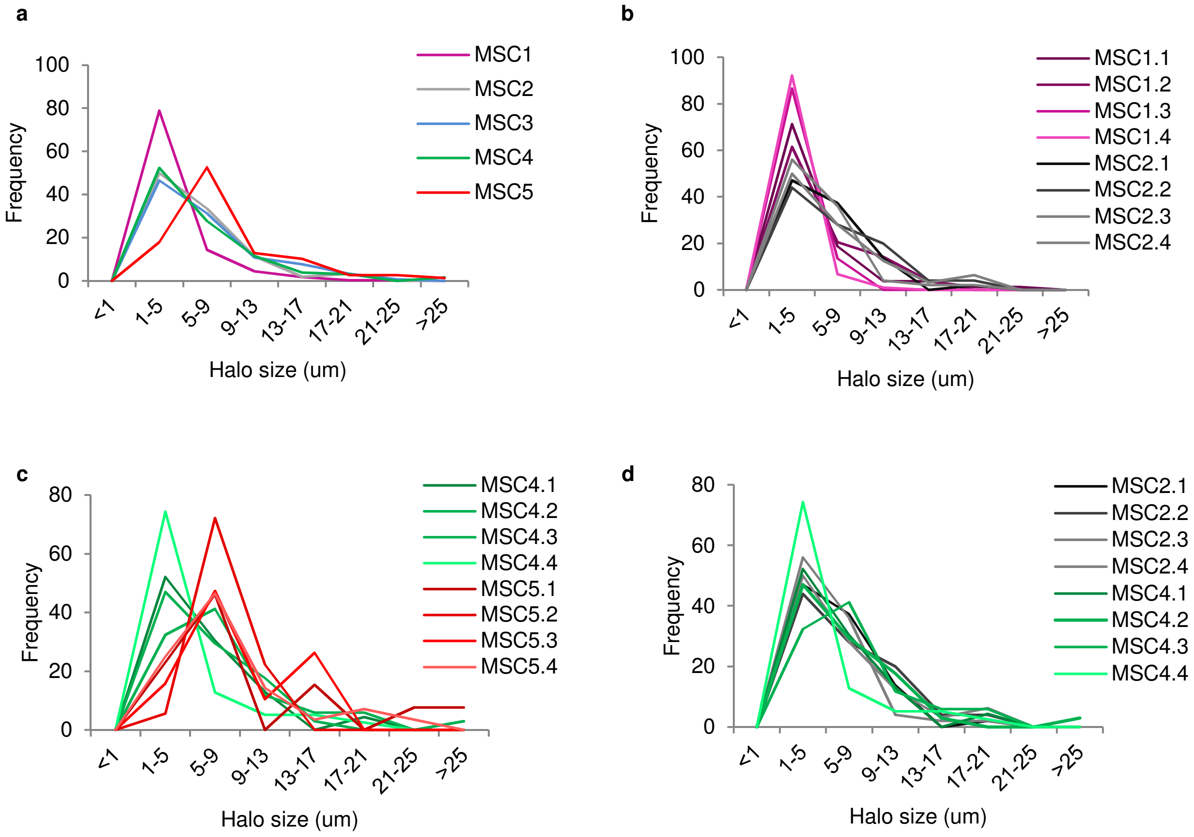

e

	One-Way ANOVA
MSCh1 - 5	$F_{(4, 853)} = 30.35, p < 0.001$
MSCh1, 3, 5	$F_{(2, 567)} = 62.34, p < 0.001$
MSCh2 - 4	$F_{(2, 442)} = 1.15, p = 0.32$
MSCh1 - 2	$F_{(1, 491)} = 43.15, p < 0.001$
MSCh2 - 3	$F_{(1, 313)} = 2.31, p = 0.13$
MSCh3 - 4	$F_{(1, 285)} = 1.05, p = 0.31$
MSCh4 - 5	$F_{(1, 206)} = 12.06, p < 0.001$

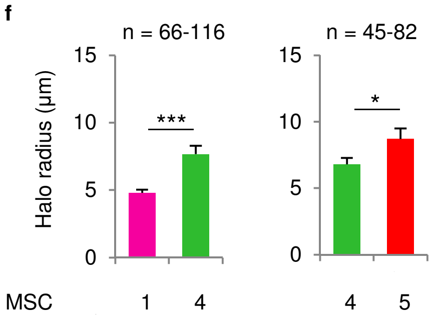

Supplementary Figure 4

Supplementary Methods - ImageJ HIM setup and validation:

RN edge determination In ImageJ, thresholds can be set based on absolute intensity values or at an intensity relative to the local maxima for each individual image. Due to the wide range of maximal intensities within a population of MFHR processed cells, an absolute pixel intensity threshold is usually not applicable to the whole population. In fact, the intensity range within an RN is more similar in a halo population than is the maximal (**Supplementary Fig. 1a**), meaning an RN threshold related to the individual maxima is useful. To set the RN threshold we compared RN radius measurements returned by HIM with different settings, to visual assessment of the RN boundary (**Fig. 1c, Supplementary Fig. 1b**), for training images.

Choice of measurements MFHR image files were analysed in ImageJ using HIM, as described in the User Guide. HIM allows separate radius measurements to be derived from the RN and the total area (**Supplementary Fig. 1f**), and therefore the calculation of a derived halo radius. Radius measurements are calculated by fitting HIM output areas to circles (User Guide). However, we note that a significant minority of entities are elliptical. Application of an elliptical formula does not significantly change halo radius measurements (not shown), so we chose to perform circle formula based calculations throughout. Circles allow the whole area to be taken into consideration rather than just x- and y-axes, which like single visual radius measurements, are more affected by structural irregularities. In most cases we choose to present our results in terms of derived halo radius, as this reflects the theoretical chromatin loop size and has biological implications. If desired, and perhaps applicable to certain specific analyses, HIM allows different types of measurements to be made quickly and easily. For example RN radius or area measurements could be used to interrogate NM volume and compaction. Note that our standard combination of settings (HIM x55/15) systematically incorporates pixels at the outer halo edge that are not visible by eye (**Fig. 1e**), meaning halo measurements are systematically larger than those generated by eye.

Effect of flare The effect of flare from a bright RN on halo measurement was assessed using unexpanded 3T3 cell halos (treated only up to 0.5 M NaCl), in which histones are not extracted[1], and chromatin remains packaged within the nucleus. HIM x55/15 returned a halo radius that is approximately 5-fold less than that created by loop expansion, indicating a small but significant, systematic contribution to halo measurements (**Supplementary Fig. 1g**).

Using HIM to determine classifications Specialist HIMs were designed which use RN intensity to non-subjectively classify halos. Populations of cells processed by MFHR return two classes of product (**Fig. 1h**). Class I have defined RNs and class II have an ill-defined RN with poor structure and fail to return a value using HIM. Class I is further divided into class Ia (bright RN) and Ib (pale RN, **Fig. 1h**). Class Ia halos are defined as those in which >50% of the returned RN from local maxima is above 220 intensity. Class Ib halos have <50% of their RN above 220 intensity. Validation is shown in **Supplementary Fig. 2a**. RN HIM was trialled with different absolute intensity values (180-250 **Supplementary Fig. 2a**) and 220 chosen. For an example training set of visually defined Ia and Ib images, HIM RNx55/RN220 returned a value of 89% for class Ia, but only 7% for class Ib. Therefore using a cut off of 50% HIM RNx55/RN220 effectively separates class Ia and Ib halos and we show that HIMRNx55/RN220 classifies the whole 3T3 dataset the same as visual scoring (**Supplementary Fig. 2a right**). Nevertheless, other cell lines are less easy to classify by eye so RN HIM provides rapid, non-subjective classification by application of a constant intensity threshold.

HIM measurements for a panel of non-cancer cell lines Application of HIM x55/15 to a panel of cell lines returned significant, reproducible differences in both halo size and proportions of class Ia, Ib and II (**Supplementary Fig. 2b-d**), implying global differences in the frequency or nature of their MARs. We considered whether changes in halo size might reflect differing proportions of classes across populations, as we find that class Ia cells return slightly larger RN radius values and slightly smaller halo radius values than Ib cells using standard HIM x55/15 (**Supplementary Fig. 2b**). However the differences we observe in halo size across cell lines is not attributable to differing distribution within class I, because separate analysis returns similar trends (**Supplementary Fig. 2c**). In fact there is not a straightforward correlation between mean halo size and classification (compare **Supplementary Fig. 2c** and **2d**). For example, NHU and MCF10A have a similar percentage of class Ia cells (78% and 88% respectively), but different mean halo radius (16 µm and 13 µm respectively), while BJ-hTERT has only 42% class Ia cells, and a mean halo size similar to MCF10A. The differences in halo parameters that we observe across a panel of non-cancer cell lines therefore shows that choice of 'normal' comparison is highly important.

Customization of HIM for MSCs Cell types respond to MFHR differently, in some cases requiring HIM thresholds to be fine-tuned for particular analyses. Mesenchymal Stem Cells (MSCs)[2] were observed to produce MFHR images with pale RNs, requiring the RN threshold criteria to be adapted so that it supports analysis across the whole diverse series. Different

RN values, based on range from local maxima (where x=55, 70, 75, or 100) were compared for measurement of MSC1 and MSC3 cell lines (**Supplementary Fig. 3b**). The HIM default threshold (x55) gave a smaller than visual measurement for both cell lines, while RN x75 gave a similar value to visual assessment (100.7% for MSC3). MSC1 RN measurements are still somewhat smaller than visual measurements at x75, which will lead to slight overestimation of halo size. In fact several sets of HIM settings were trialled on the MSC series data (**Supplementary Fig. 3c**). HIM x75/x180 (**Fig. 2b**) was used to derive a halo measure in which both parameters are related to RN intensity. For this analysis, the range settings for outer threshold (x180) was chosen to support inclusion of a greater proportion of cells across the series. Outer range settings higher than 180 incorporate smaller percentages of the population, but still report differences in halo measurements between MCS1 and 3 (**Supplementary Fig. 3c, d)**. A different version of HIM (x75/15, **Fig. 2c**) uses an outer threshold with absolute pixel intensity of 15, which reflects the point at which intensity increases over background, where background is set at 5% AUC for a training population (**Fig. 1e**). Here outer threshold is unaffected by RN intensity and reports only on differences in RN and halo size. We remind readers that data collected using different thresholds should not be compared. For example, mean halo measurements in figure 2c cannot be directly compared to figure 2b, except to evaluate the effect of the different settings.

Customisation of classification HIM for MSCs To establish suitable parameters for classification of MSCs by HIM, data collected using different absolute RN pixel intensity thresholds were compared to classifications by eye for a representative set of images for MSC1 (**Supplementary Fig. 3e, f**). Those that were visually scored as class Ia, but by HIM were scored as class Ib, are defined as false Ib, and those visually scored as Ib, but by HIM scored as Ia, are defined as false Ia. For MSC1, classification based on a 50% cut off, using intensity above 180 or 200 caused significant percentages of false Ia images. Likewise, above 230 produced a significant percentage of false Ib images (**Supplementary Fig. 3f**). We chose 220 intensity as our parameter, as this minimized ‘false’ classification with the training set, and confirmed that this is suitable across the full range of the MSC series (**Supplementary Fig. 3g**), before application to test populations.

Validation of biological differences As with all analysis methods that image slides prepared in different sessions, we considered whether biological differences could be seen above the level of variability between replicate experiments. To illustrate this, frequency distributions were plotted for halo size measurements generated using HIM x75/x180 from MSC cell lines, for complete data sets (**Supplementary Fig. 4a)** and individual biological

and technical replicates (**Supplementary Fig. 4b, c, d**), and variance calculated using ANOVA (**Supplementary Fig. 4e)**. Graphs show that individual replicates from the same cell line overlay well and are more similar to each other than to data sets from a different cell line, compare MSC1 and 2 (**Supplementary Fig. 4b**) and MSC4 and 5 (**Supplementary Fig. 4c**). Furthermore, individual data sets return the same results as the mean of each set of replicates (**Supplementary Fig. 4a, Fig. 2b**). ANOVA results confirm that MSC1 halo size data set is statistically different from MSC2, and that MSC4 is statistically different from MSC5, but MSC2 is not different to MSC4. Overall, this shows that variation inherent in preparation conditions is far less than biological differences between cell lines.

In addition, we processed and imaged MSC1/MSC4 and MSC4/MSC5 as pairs, in order to ensure identical reagents and process. As expected, we find the same statistical differences as when processed independently (compare **Supplementary Fig. 4f** and **Fig. 2b**). Taken together this allows us to conclude that the differences we observe between cell lines is not due to slide to slide variability but statistically robust biological differences due to cell type.

Supplementary Table 1: Versions of HIM, with their applications

**HIM title**	**Usage**
HIM x55/15	Halo size for non-cancer and cancer cell lines<br>Defines RN (local maxima less 55) and outer threshold (intensity 15) areas
HIM x75/15	Halo size for MSC series<br>Defines RN (local maxima less 75) and outer threshold (intensity 15) areas
HIM x75/x180	Halo size for MSC series (includes effect of RN intensity)<br>Stability for MSC series<br>Defines RN (local maxima less 75) and outer threshold (local maxima less 180) areas
HIM x55/x240	Stability for non-cancer and cancer cell lines<br>Defines RN (local maxima less 55) and outer threshold (local maxima less 240) areas
HIM RNx55/RN220	Classification for non-cancer and cancer cell lines<br>Defines the RN percentage above 220 intensity
HIM RNx75/RN220	Classification for MSC series<br>Defines the RN percentage above 220 intensity

User Guide: Halo Image Macro for ImageJ

File output HIM opens each image file, selects a region of interest (ROI) within each image based on the ‘RN threshold’, measures the RN area and saves a copy of the new file, with the ROI shown, in the ‘RN threshold’ folder. This is repeated for the ‘outer threshold’, saving the new file in the appropriate folder. The ‘setBatchMode’ command in the HIM code stops the visual opening and processing of each file. This command may be removed to visualize the steps and allow troubleshooting.

The output for each file is a RN threshold analysis picture, an outer threshold analysis picture, and area measurements for each. Measurements are initially recorded in the ‘Results’ dialogue box. HIM then matches RN and outer area measurements for each cell and exports the data to a .csv (comma-separated values) file which can be read by excel. Users should then save this file as a .xlsx file or other excel file type as some excel features are not compatible with .csv files.

Matching RN and outer measurements RN and outer area measurements from the same cell are matched by using x- and y- positional information for the centre of each ROI. The HIM queries a possible pair (from within the same image) and if the x- and y- centre coordinates of the RN and outer differ by less than 5% of the square root of the total image area, measurements are linked with the outer data moved to the same row as the RN data. If the pair fail to fulfil this criteria, HIM will query any other RN measurements from the image (ie if multiple cells are present on the image) to see if they match with the outer measurement. If no matching RN is found, the outer measurement data are not moved and they remain on their own row of the output .csv file. HIM is therefore capable of dealing with images with multiple cells which may or may not record both an RN and outer measurement and which may not be recorded in the same order. However, users are advised to prepare slides using a cell plating concentration that does not result in excessive cells per image as it is more likely that cells will be touching. HIM cannot discriminate a pair of touching cells from one single cell and images should be quickly checked for any such occurrences.

In addition, when HIM has finished processing each file within the source folder, any images where a RN and outer pair was not found for all ROIs will be listed in a dialogue box titled, "Files requiring manual curation". Images will also be listed here for which no RN, or no outer was detected.

Images which 'fail' HIM also checks any outer measurements which 'fail' (see description of stability measurements and class II cells) in that the outer measurement occupies more than 80% of the image area. This happens typically for pale cells when outer measurements are measured using the local maxima less x method eg when unstable cells decay during stability analysis. Outer measurements that are larger than 80% of the image area are replaced by 'fail' under outer area measurement heading. This is applied to all RNs on an image for which an outer area measurement is classified as a fail.

Results column headings For cells with a matched RN and outer measurement, the cell returns recordings under the following headings;

Label - this is the image name followed by RN indicating a unique RN
Area - RN area
X - central x- position of RN ROI - used for positional information
Y - central y- position of RN ROI - used for positional information
Image No. - this refers to the numbered RN on the RN threshold analysis image saved in the 'RN' folder and maybe used to match cell data back to the original image
Outer Area - matching outer area measurement
Outer X - central x-position of outer ROI - used for positional information
Outer Y - central y-position of outer ROI - used for positional information
Outer Image No. - this refers to the numbered outer on the outer threshold analysis image saved in the 'Outer' folder. Note: outers from different images may have the same number, this is due to results being recorded and then being moved to the appropriate RN row. Therefore, if wishing to refer back to outer threshold images users should check the image name and outer number carefully.

Headings are slightly different for classification HIM output with outer replaced by absolute RN. This refers to the ROI generated using an absolute pixel intensity threshold rather than one related to the local image maxima.

To run HIM:

- Install ImageJ from http://rsb.info.nih.gov/ij/download.html. HIM is compatible with ImageJ for Mac OS X or Windows, version 1.47o or later. Previous versions do not accept strings in the Results table data columns (for example, used here for ‘fail’).

- Download appropriate HIM .txt file from supplementary files, into a dedicated folder. If desired, replace default threshold settings (x55/15), chosen after calibration to your cell type.

- Create a source folder for MFHR images that are to be analysed, and three destination folders for 'RN threshold' and 'outer threshold' respective output pictures and a folder for excel readable files.

- Populate source folder with a set of MHFR image files. We use ‘TIFF For Publication’ files, derived from Openlab image acquisition software for Mac, however HIM will analyse any high resolution ImageJ compatible file types. Please note, the first image to be analysed must contain at least one cell otherwise HIM will not return any information for that image. Images without a cell are tolerated after the first image and the HIM will return *no RN and *no outer for such images.

- Open ImageJ and load HIM with the command:
  Plugins –
  Macros –
  Install...
  Then select the appropriate HIM .txt file.

  Run HIM in ImageJ with the command:
  Plugins –
  Macros -
  then select the HIM you have just loaded.

  When measuring different sets of images with the same HIM, the HIM file only needs loading at the beginning of the session. However, if changing between different HIMs then they will need to be reloaded each time.

- After selecting the HIM to run, a dialogue box opens. Select the appropriate source folder, then when prompted, select the RN and outer threshold destination folders. Finally, select the results folder and type your desired name for the .csv results file. HIM then runs without further prompting.

- When HIM is running, the ImageJ results table is visible. However, once the HIM saves these data into the .csv file the results table closes. On completion of HIM, a

dialogue box entitled "Files requiring manual curation" appears highlighting any files that proved difficult to analyse, either due to *no RN or * no Outer being measured, or if pairs of RN and outers could not be linked. Upon completion the user should check these images and respective RN and outer threshold analysis files and adjust the .csv file accordingly. Output images should also be checked for joined cells, cells cut by the edge of the picture and occasional extraneous particles which should all be removed. This can also be done at the image acquisition stage, though random acquisition is preferred.

- Calculate halo radius using the formula;

  Halo radius = $\left\{\left(\frac{\sqrt{'outer\ area'}}{\pi}\right) - \left(\frac{\sqrt{'RN\ area'}}{\pi}\right)\right\} \times c$

  where c is a conversion factor between pixels and μm

  Determine conversion factor for your imaging equipment and software using automated scale bar functions, or directly by imaging a graticule under the same conditions used to image halos. Calibration between pixels and micrometers should be made following consistent file transfer protocols, as pixel measurements are not always consistent between software.

  Micrometer measurements can be converted to an estimate of chromatin loop size in kilobases using conversion factors published previously. These range from 1 μm = 3 kbp[3,4], to 1 μm = 2.3 kbp[5]. We chose to use the former, based on 10.4 bp per helical turn in B-form DNA[6] with a helical turn of 3.4 nm[7]. Note that radius values should be doubled to reflect the fact that loops are composed of two radii.

These steps take about 5 minutes per coverslip including ~1 minute of macro running time.

Analysis specific to classification The classification HIM generates the standard RN area measurement based on local maxima and a second RN area based on an absolute intensity threshold, from which are % is derived. If this is above 50%, the RN is designated as class Ia, if this is equal to or below 50%, the RN is designated as class Ib.

Analysis specific to stability measurements Data from stability measurements can be presented as time to fail, or percentage of surviving halos as described earlier.

Manual execution Instructions for manual execution of the steps carried out automatically by HIM x55/15 are detailed below (direct instructions in italics). Where both thresholds are relative to the local maxima (for example HIM x55/x240), outer threshold is measured in the same way as RN using appropriate threshold. This does not include the matching of RN and outer measurements which can be found in the HIM .txt file.

Open file in ImageJ

Select ImageJ: Image: Type: 8-bit

This converts the image to greyscale 8-bit values from RGB.

ImageJ: Process: Find maxima

Dialogue box opens, *select Noise tolerance 55, Maxima within tolerance, nothing ticked*. This results in a new picture, and selects the pixels within a tolerance of 55 from the local maximal intensity of the image. The area in white will be measured.

ImageJ: Process: Binary: Close

The individual pixels selected will then be closed as an ROI.

ImageJ: Image: Adjust: Threshold

In Threshold dialogue box, *Set thresholds at 255, default, red, box not ticked, Apply, close box*

ImageJ: Analyze: Analyse Particles

In dialogue box, *Size 3000-Infinity, Pixel units ticked, Circularity 0.00-1.00, Show Outlines, Only Display results and Exclude on Edges ticked*.

This results in area measurements taken for white areas bigger than 3000 pixels.

Save new picture in Threshold RN, Close all open files except raw image.

To measure outer threshold area…

Image: Adjust: Threshold…

In Threshold dialogue box, *Set,* in Set Threshold Levels dialogue box *Lower Threshold Level at 15, Upper Threshold Level at 255, ok*. In Threshold dialogue box, *default, red, Dark background ticked, apply, close box.* In NaN Background dialogue box *ensure Background Pixels to NaN is not ticked, ok.*

Note that this results in an absolute pixel intensity threshold unlike that for RN.

ImageJ: Process: Binary: Convert to Mask

ImageJ: Process: Binary: Close-

ImageJ: Analyze: Analyze Particles

In dialogue box, Size 3000-Infinity, Pixel units ticked, Circularity 0.00-1.00, Show Outlines, Only Display results and Exclude on Edges ticked.

Save new picture in Threshold Outer, Close all open files.

Technical tips for MFHR processing and image generation

These are provided to help minimize other sources of variability.

- The pH of buffers should be checked carefully as this can affect halo stability
- Buffers should be made up in advance and stored in aliquots at -20ºC, to generate complete data sets with one batch. Working aliquots may be kept at 4°C for one week
- Buffers should be pre-cooled on ice, and all processing performed on ice
- Process and image one coverslip at a time
- Carefully lower coverslips into solutions to prevent loss of cells. Do not pipette buffers onto coverslips.
- Standardise distance from cells to UV light source
- Seal mounted coverslips with clear nail varnish to prevent buffer flow
- Keep slides in the dark during the development period
- Collect images within a limited time period (we use 10 minutes) to prevent analysis of degraded halos.
- Minimize time between visualization of a halo and image capture
- Take care not to re-image areas of the coverslip that have already been exposed to microscope light.
- For class analysis no halo should be excluded from image capture. These image sets can also be used for size analysis. If only size analysis will be performed, joining halos can be excluded at image capture, since HIM cannot measure these halos.
- Exposure time, and RN and outer thresholds should be set using a training set.

References for supplementary material

1 Hesketh, E. L., Knight, J. R. P., Wilson, R. H. C., Chong, J. P. J. & Coverley, D. Transient association of MCM complex proteins with the nuclear matrix during initiation of mammalian DNA replication. *Cell Cycle* **14**, 333-341, doi:10.4161/15384101.2014.980647 (2015).

2 Funes, J. M. *et al.* Transformation of human mesenchymal stem cells increases their dependency on oxidative phosphorylation for energy production. *Proc Natl Acad Sci U S A* **104**, 6223-6228, doi:10.1073/pnas.0700690104 (2007).

3 Buongiorno-Nardelli, M., Micheli, G., Carri, M. T. & Marilley, M. A relationship between replicon size and supercoiled loop domains in the eukaryotic genome. *Nature* **298**, 100-102 (1982).

4 Vogelstein, B., Pardoll, D. M. & Coffey, D. S. Supercoiled loops and eucaryotic DNA replicaton. *Cell* **22**, 79-85 (1980).

5 Lemaitre, J. M., Danis, E., Pasero, P., Vassetzky, Y. & Mechali, M. Mitotic remodeling of the replicon and chromosome structure. *Cell* **123**, 787-801, doi:10.1016/j.cell.2005.08.045 (2005).
6 Wang, J. C. Helical repeat of DNA in solution. *Proc Natl Acad Sci U S A* **76**, 200-203 (1979).
7 Watson, J. D. & Crick, F. H. Molecular structure of nucleic acids; a structure for deoxyribose nucleic acid. *Nature* **171**, 737-738 (1953).
